# Iterative Restoration of the Fringe Phase (REFRASE) for QSM

**DOI:** 10.3389/fnins.2021.537666

**Published:** 2021-05-13

**Authors:** Johannes Lindemeyer, Wieland A. Worthoff, Aliaksandra Shymanskaya, N. Jon Shah

**Affiliations:** ^1^Institute of Neuroscience and Medicine–4, Forschungszentrum Jülich, Jülich, Germany; ^2^Institute of Neuroscience and Medicine–11, Forschungszentrum Jülich, Jülich, Germany; ^3^JARA–BRAIN–Translational Medicine, Aachen, Germany; ^4^Department of Neurology, Rheinisch-Westfälische Technische Hochschule (RWTH) Aachen University, Aachen, Germany

**Keywords:** magnetic resonance imaging, phase imaging, field mapping, background field removal, quantitative susceptibility mapping

## Abstract

In quantitative susceptibility mapping (QSM), reconstructed results can be critically biased by misinterpreted or missing phase data near the edges of the brain support originating from the non-local relationship between field and susceptibility. These data either have to be excluded or corrected before further processing can take place. To address this, our iterative restoration of the fringe phase (REFRASE) approach simultaneously enhances the accuracy of multi-echo phase data QSM maps and the extent of the area available for evaluation. Data loss caused by strong local phase gradients near the surface of the brain support is recovered within the original phase data using harmonic and dipole-based fields extrapolated from a robust support region toward an extended brain mask. Over several iterations, phase data are rectified prior to the application of further QSM processing steps. The concept is successfully validated on numerical phantoms and brain scans from a cohort of volunteers. The increased extent of the mask and improved numerical stability within the segmented *globus pallidus* confirm the efficacy of the presented method in comparison to traditional evaluation.

## 1. Introduction

Since emerging as a novel magnetic resonance imaging (MRI) contrast less than a decade ago, quantitative susceptibility mapping (QSM) has rapidly evolved in terms of evaluation strategies and applications, exploiting the magnetic properties of tissue within the brain based on the phase information (Schenck, 1996; Reichenbach, 2012; Duyn and Schenck, 2017; Eskreis-Winkler et al., 2017). The foundation of any successful phase processing scheme is, however, defined by the quality of the phase data and, as a direct consequence, by the extent of the exploitable data support (Elkady et al., 2016). Being an ill-conditioned, spatially convolved problem, QSM requires a continuous volume for evaluation. As three-dimensional deconvolution is the central step of QSM, even small areas of incorrect phase or field information at the rim of the volume of interest, typically defined by a mask, will strongly propagate errors into the entire susceptibility map. This can obscure or impair the contrast of small anatomic details (e.g., Fortier and Levesque, 2018).

Other than a small number of approaches that aim to directly evaluate the complex signal (e.g., Liu et al., 2013; Wang and Liu, 2015), most algorithms initially unwrap the acquired phase in both the space and time domains or only in the time domain (e.g., de Rochefort et al., 2010; Liu et al., 2011b; Schweser et al., 2011, 2016; Sun and Wilman, 2015). Unwrapping is, in general, only possible when a continuous phase function describes the true data. Furthermore, the signal-to-noise ratio (SNR) should be high enough to represent this function and the true phase change per voxel or time point must remain below *π*, fulfilling the Nyquist-Shannon criterion. Susceptibility contrast between brain tissue and surrounding areas, such as blood vessels, bone, or air, can create strong local field distortions (Schenck, 1996; Liu et al., 2015; Dixon, 2018; Fortier and Levesque, 2018), These distortions can generate undersampling in the time domain, whenever the relative field shift is strong enough, and in the spatial domain, when the phase gradients between voxels exceed the Nyquist criterion. Areas within the measured volume that are affected by any of the aforementioned impairments will be referred to as EPRs in the following and have to be excluded from the evaluation area (EA) by masking or a weighting step prior to data processing. The focus of this study is on EPRs that do not contain extreme susceptibility shifts or signal voids and are only undersampled due to strong gradients of external origin.

In healthy subjects, strong sources of field distortions, known as background fields, tend to reside outside of the brain and their influences on QSM are identified and addressed by tailor-made background field removal strategies (e.g., Shmueli et al., 2009; Liu et al., 2011a; Schweser et al., 2011; Sun and Wilman, 2013; Lindemeyer et al., 2015). Most background field removal approaches determine the nature of the background fields based on physical principles such as harmonic distortions (e.g., Schweser et al., 2011; Wu et al., 2012; Topfer et al., 2015; Özbay et al., 2017) or dipole fields (e.g., de Rochefort et al., 2010; Liu et al., 2011a). Only a few approaches (such as Neelavalli et al., 2009; Wharton et al., 2010) exist that quantify the value and position of actual dipole sources, rather than a pseudo-susceptibility distribution, as this step is not required for the correction of the EA. Some approaches, such as SHARP (Schweser et al., 2011), even reduce the spatial coverage of the EA, depending on the applied filter kernel. In a related approach, Wen et al. (2014) make use of iterative optimisation to improve the accuracy of the background field fitting. Laplacian approaches, such as that from Zhou et al. (2014), remove background influences and, at the same time, can potentially unwrap the raw phase. Nevertheless, their reliability is uncertain, especially when facing strong local contrast and undersampling (Fortier and Levesque, 2018). An extensive discussion on the underlying theory of various approaches for background field removal has been published by Schweser et al. (2017).

In contrast to correcting estimated field maps after unwrapping, our novel approach aims to retrospectively correct the phase within the EPRs in an iterative manner based on the obtained background field correction. Previously published iterative approaches, such as Buch et al. (2014), have successfully modelled air and bone contributions to improve the quality and the coverage of susceptibility maps. In contrast, we address arbitrary sources of field distortion without the need for discrete source localisation. The presented algorithm, REstoration of the FRinge phASE (REFRASE), makes use of the employed background field correction within the EA to obtain additional information on global background fields. In the present study, we apply this novel concept to the harmonic and dipole field corrections obtained with MUltistage BAckground FIeld REmoval (MUBAFIRE) (Lindemeyer et al., 2015). These corrections are modified to produce approximative background field maps for the erroneous phase region (EPR) based only on data from within the EA. With this information, the measured phase can be iteratively corrected before further processing takes place, ideally recovering data from the EPRs and hence extending the overall EA.

**Figure 1:**
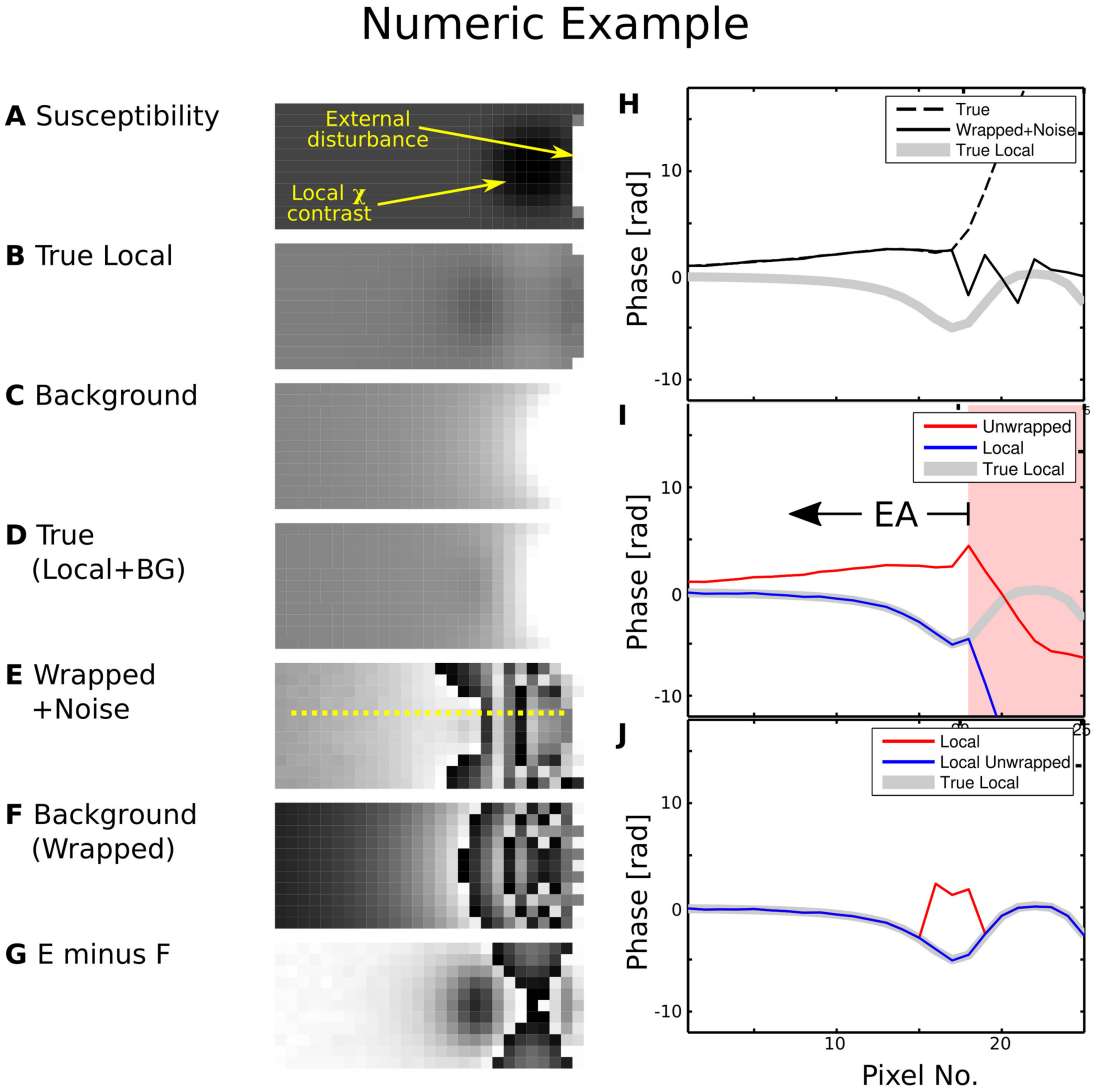
Numeric phase data example. The left-hand side shows **(A)** a susceptibility map, **(B)** the local phase generated by the smooth susceptibility sphere from **(A,C)** background phase generated by the external disturbance, **(D)** True composed phase **(B)+(C)**, **(E)** “Measurement” simulated by wrapped phase **(D)** with noise contamination, **(F)** Wrapped true background phase **(C,G)** background phase **(F)** subtracted from measurement **(E)**. The yellow line cut indicated in **(E)** was processed in **(I)** and **(J)**. The right-hand side shows plots along the line cut for **(H)** true phase, wrapped phase with noise contamination and true local phase, **(I)** first unwrapped, then background-subtracted phase, **(J)** first background-corrected as in **(G)**, then unwrapped phase. Greyscale colour ranges are: **(A)** [−6, 15] ppm, **(B–D)** [−20, 20] rad, **(E–G)** [0, 2*π*] rad. Prior background phase removal **(J)** leads to a correct local phase estimation for pixels beyond no. 18, while first performing the unwrap does not [area with background shaded in red in **(I)**]. For case **(I)**, the EA would have to be restricted to voxels left of no. 18.

The difference between field mapping applied to a dataset with and without prior phase correction is illustrated in Figure 1. Erroneous unwrap results are triggered when the measured phase is directly employed, biasing the estimate of the background field. Hence, the estimated local field strongly deviates from the true local field for pixels beyond the EPR (Figure 1, pixel 18). This makes evaluation of the local contrast (blurry black spot in the image), e.g., for QSM, impossible.

Since QSM maps are based on a non-local deconvolution of the corrected field map, structures near the surface of the EA and contrast in its centre suffer from partial data coverage (missing data) or regions with incorrectly estimated field values. By offering partial data recovery, our REFRASE method, first outlined in the form of a prototype in Lindemeyer et al. (2017), enables QSM with a greater coverage and enhances the reconstruction quality of QSM maps within the EA. Here, the feasibility and quality of REFRASE are assessed with numerical samples and via the homogeneity within segmented anatomical structures in a cohort of *in vivo* subjects.

## 2. Materials and Methods

### 2.1. Theory

#### 2.1.1. Multidimensional Unwrapping

Let *φ*[*i*] be the true and $\hat{\varphi }\left[i\right]$ be the measured phase in discrete, evenly-spaced positions $\vec{r}\left[i\right]$ along a path *i* = 0, . . . , *n*. The transformation $\hat{\varphi }\left[i\right]=\text{mod}\left(\varphi \left[i\right],2\pi \right)$ can only be inverted, if the condition |*φ*[*i* + 1] − *φ*[*i*]| ≤ *π*, describing the Nyquist limit, holds for every pair (*i*, *i* + 1). This inversion is commonly known as the *unwrapping* operation and will be denoted by $\varphi \left[i\right]=U\left(\hat{\varphi }\left[i\right]\right)$. For multidimensional datasets, this condition must hold for at least one path between two locations to render these mutually unwrappable. The set can only be entirely unwrapped if such a path exists for every pair of voxels within a (multidimensional) set of voxels. Depending on the unwrapping strategy, additional conditions might apply.

#### 2.1.2. Data Masking

Whilst the true phase change is well-known in synthetic data, it remains unknown in measured data as it is concealed by the acquisition process. Generally speaking, even a measured phase dataset, in which phase wraps are spaced wider than the Nyquist limit of two voxels, can originate in a phase profile that was measured with significant undersampling, meaning that the actual phase wrap distance is much smaller than the limit (e.g., Dagher et al., 2014). Consequently, the resulting unwrap would significantly underestimate the true slope. An MRI measurement of a human subject acquired with reasonable parametrisation normally contains a region where unwrapping in time is possible and where all true phase gradients lie below the Nyquist limit. With increasing distance from this region, which typically resides in the geometric centre of the imaged volume and near the magnet's isocentre, field, and thus phase homogeneity, decreases due to global and local perturbations. Additionally, the SNR drops rapidly within strong field perturbations or when leaving the support of a continuous object.

Data fidelity has to be assessed based on the observed phase. If the slope of a function exceeds the Nyquist limit at a certain point, its gradient will first approach *π*/Voxel, leading to a strong dephasing of the local neighbourhood of the observed point at position [*ijk*] in the spatial domain. This can be evaluated by the so-called local coherence (LC) metric as described by Witoszynskyj et al. (2009):

(1) $$\begin{array}{l} Q_{\text{LC}}\left[ijk\right]=\frac{1}{27}\overset{i+1}{\underset{s_{i}=i-1}{\sum }}\overset{j+1}{\underset{s_{j}=j-1}{\sum }}\overset{k+1}{\underset{s_{k}=k-1}{\sum }}\text{exp}\left(\text{i}\hat{\varphi }\left[s_{i}s_{j}s_{k}\right]\right) \end{array}$$

The presence of noise will further diminish *Q*_LC_, making the LC a good estimator for data validity and hence for delineating the EA. In order to avoid non-random noise bias in the near neighbourhood of 27 voxels, the LC can be smoothed, e.g., by a narrow-width Gaussian, e.g., with *σ* = 2 voxels, prior to thresholding.

The thresholding limit, *Q*_LC_ ≥ *Q*_LC,min_, is initially determined heuristically. In order to achieve reasonable masking, one has to consider the employed static magnetic field strength, voxel dimensioning, sequence parametrisation, and the evaluated echo time. Furthermore, one must also consider acquisition-specific artefacts such as fold-ins, regions of low signal or phase poles. For a given experimental setup, *Q*_LC,min_ can be optimised for best result fidelity based on processing a cohort of measurements with several parametrisations.

#### 2.1.3. Field Separation

Let *b* = *b*[*i*] = *φ*[*i, t*_TE_]/2*πt*_TE_ be a function along a discrete path in space, indexed by *i* = 1, . . . , *n*. Without loss of generality, we neglect phase offsets in this description (⇒ *φ*[*i*, 0]: = 0). Let further *b* be composed of a dominant function *b*_bg_ and a local characteristic *b*_local_, with 2*πt*_TE_·|*b*_local_[*i*] − *b*_local_[*i* + 1]| ≤ *π* ∀*i*. Omitting the path index, this gives the commonly used separation (e.g., Deistung et al., 2017):

(2) $$\begin{array}{l} b=b_{\text{bg}}+b_{\text{local}} \end{array}$$

It is clear that:

(3) $$\begin{array}{l} {\text{mod}}_{2\pi }(2\pi t_{\text{TE}}\cdot b_{\text{local}})={\text{mod}}_{2\pi }(2\pi t_{\text{TE}}\cdot (b-b_{\text{bg}}))\\ \;={\text{mod}}_{2\pi }({\text{mod}}_{2\pi }(2\pi t_{\text{TE}}\cdot b)\\ \;-{\text{mod}}_{2\pi }(2\pi t_{\text{TE}}\cdot b_{\text{bg}})) \end{array}$$

While *b*_local_ can be unwrapped in a local neighbourhood, *b* and *b*_bg_ may exceed the Nyquist limit. Further, $\hat{\varphi }\left(t_{\text{TE}}\right)={\text{mod}}_{2\pi }\left(2\pi t_{\text{TE}}\cdot b\right)$ is the measured phase and ${\tilde{b}}_{\text{bg}}$ is an estimate for *b*_bg_. The local phase estimate can be derived from the difference:

(4) $$\begin{array}{l} {\tilde{\varphi }}_{\text{local}}\left(t_{\text{TE}}\right)={\text{mod}}_{2\pi }\left(\hat{\varphi }\left(t_{\text{TE}}\right)-{\text{mod}}_{2\pi }\left(2\pi t_{\text{TE}}\cdot {\tilde{b}}_{\text{bg}}\right)\right)\;. \end{array}$$

Now, ${\tilde{\varphi }}_{\text{local}}$ can be unwrapped, leading the field map ${\tilde{b}}_{\text{local}}$:

(5) $$\begin{array}{l} {\tilde{b}}_{\text{local}}=\frac{U\left({\tilde{\varphi }}_{\text{local}}\right)}{2\pi t_{\text{TE}}}\;. \end{array}$$

Reinserting the previously subtracted background field estimate, the true field distribution is recovered. Including (2), (4), and (5), this leads to the comprehensive term:

(6) $$\begin{array}{l} b={\tilde{b}}_{\text{bg}}+\frac{U\left({\text{mod}}_{2\pi }\left(\hat{\varphi }\left(t_{\text{TE}}\right)-{\text{mod}}_{2\pi }\left(2\pi t_{\text{TE}}\cdot {\tilde{b}}_{\text{bg}}\right)\right)\right)}{2\pi t_{\text{TE}}} \end{array}$$

A region can be entirely unwrapped if ${\tilde{b}}_{\text{bg}}$ can be estimated everywhere within and if each pair of member points can be connected via a discrete path $\vec{r}\left[i\right]$, indexed by *i* = [1, . . . , *n*], that lies inside the region.

### 2.2. Methods

#### 2.2.1. REFRASE Optimisation Concept

Equation (6) only holds in regions where the argument in the numerator fulfils the Nyquist criterion, i.e., where ${\tilde{b}}_{\text{bg}}$ is a sufficient approximation. The *b*_bg_ within EPRs can only be estimated based on background field removal within the EA, where data fidelity is provided by prior phase coherence thresholding. This motivates the use of an iterative correction scheme working to grow the reliable EA in each step.

For phase data, $\hat{\varphi }$, of a typical multiple-echo gradient-echo measurement, let *m*_max_ be the maximum achievable brain mask and *m*_EA_ ⊆ *m*_max_ be a valid EA that can be fully unwrapped. A contiguous EA can be segmented via the process of determining the local phase coherence *Q*_LC_ as in Equation (1) and through the removal of disjoint regions. To compensate for the low phase SNR at early echo times, a Gaussian filter (*σ* = 2 voxels) is applied to the first echo before field mapping. Subsequently, the field map calculated from $\hat{\varphi }$ is expected to be valid within *m*_EA_ and allows the background field to be estimated.

Following these steps, harmonic and dipole-shaped fields are determined by MUBAFIRE (Lindemeyer et al., 2015). The harmonic result is composed from a superposition of orthonormalised solid spherical harmonic functions. As the ${\tilde{b}}_{\text{SSH}}$ is only dependent on the harmonic order and the coefficients, it can be calculated within the expanded region *m*_max_. Dipole fields are usually estimated via a minimisation term within the EA and originate from its complement, not(*m*_EA_) (e.g., de Rochefort et al., 2010). An additional condition, preventing field sources within *m*_max_, is introduced, resulting in the minimisation expression:

(7) $$\begin{array}{l} \underset{\chi_{\text{ext}}}{\min}∥m_{\text{EA}}\left[b-B_{0}\left(\chi_{\text{ext}}*d\right)\right]{∥}_{2}^{2}+\lambda ∥m_{\text{max}}\cdot \chi_{\text{ext}}{∥}_{2}^{2}\;, \end{array}$$

where *B*_0_ is the static magnetic field, *d* is the dipole kernel (de Rochefort et al., 2010), *χ*_ext_ is the external susceptibility distribution and * is the spatial convolution operation. The Tikhonov regularisation suppresses susceptibility sources inside *m*_max_. In order to avoid strong streaking artefacts at the volume surfaces, morphologic dilation can be used such that *m*_max_ extends *m*_EA_ by at least one voxel. Using the previously determined *χ*_ext_, the externally generated dipole background field for *m*_max_, ${\tilde{b}}_{\text{DIP}}$ is calculated using

(8) $$\begin{array}{l} {\tilde{b}}_{\text{DIP}}\approx B_{0}\cdot \left(\chi_{\text{ext}}*d\right)\cdot m_{\text{max}} \end{array}$$

Strong edge artefacts can be avoided by Gaussian smoothing of ${\tilde{b}}_{\text{DIP}}$ with *σ* = 1 voxel

**Figure 2:**
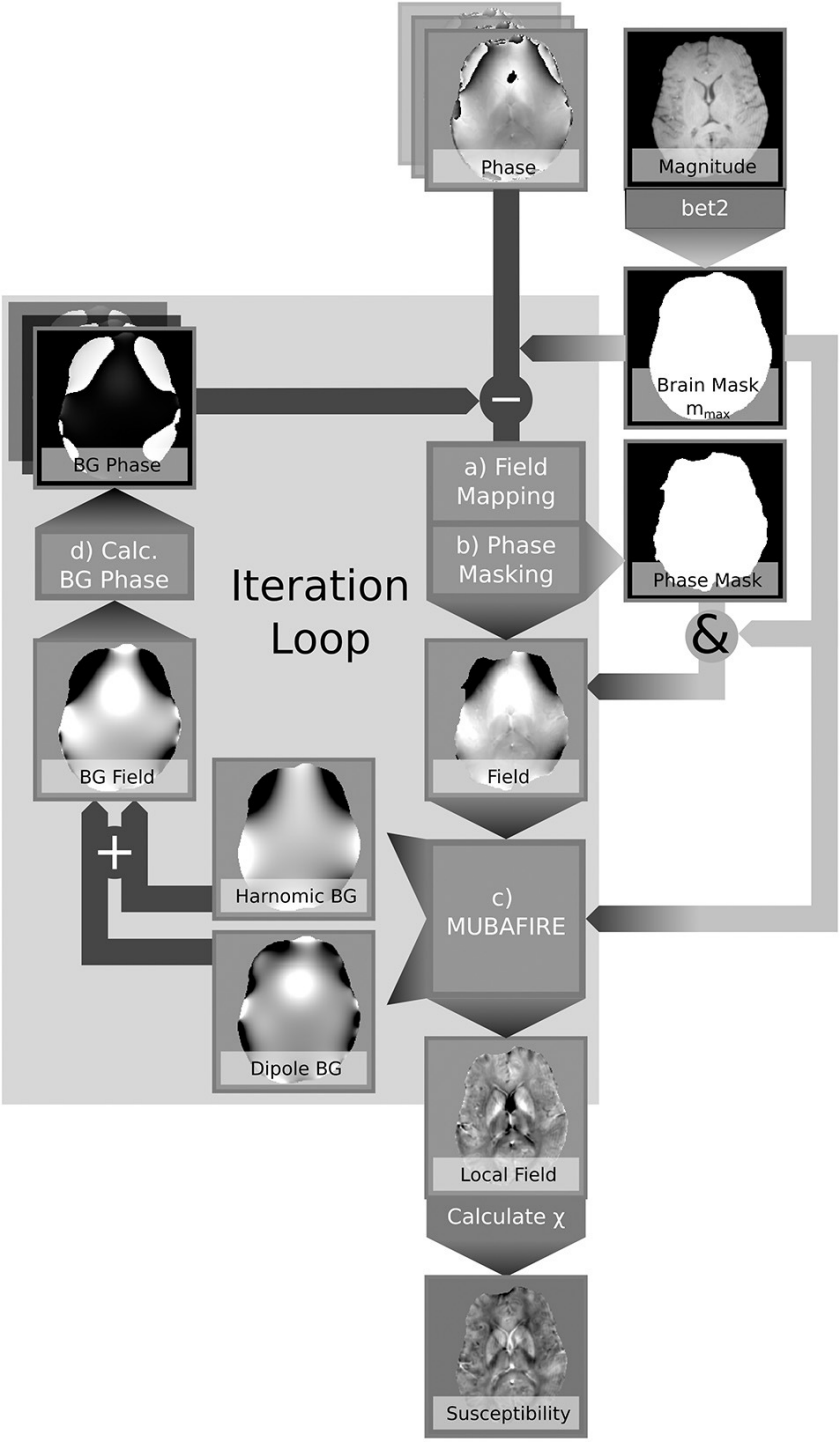
Processing scheme illustrating the iterative correction applied by REFRASE.

Summing up both components, the background phase derived from ${\tilde{b}}_{\text{bg}}={\tilde{b}}_{\text{SSH}}+{\tilde{b}}_{\text{DIP}}$ is subtracted from the original phase using Equation (4). The processing pipeline is illustrated in Figure 2 and reads:

1. Iterative correction loop (step counter *j*, ${\tilde{b}}_{\text{bg},0}:\;=\;0$, *φ*_bg, 0_ = 0)
  (a) **Field Mapping:** Spatial unwrapping of the phase in each echo (Abdul-Rahman et al., 2007), ${\text{mod}}_{2\pi }\left(\hat{\varphi }-\varphi_{\text{bg}}\right)$ within *m*_max_; temporal unwrapping in a central reference point; linear regression.
  (b) **Phase Masking:** $m_{\text{EA}}=\left[Q_{\text{LC}}\left({\text{mod}}_{2\pi }\left(\hat{\varphi }-\varphi_{\text{bg}}\right)\right)\geq Q_{\text{LC},\text{min}}\right]$
  (c) **Background Field Correction:** Identification of spherical and dipole shaped field distortions within *m*_EA_ using MUBAFIRE Lindemeyer et al. (2015); expanding to *m*_max_; $\Rightarrow {\tilde{b}}_{\text{bg},j}$ (Equation 4).
  (d) (re-)calculate **background phase** within *m*_max_:
    (9) $$\begin{array}{l} {\tilde{b}}_{\text{bg}}={\tilde{b}}_{\text{bg}}+{\tilde{b}}_{\text{bg},j} \end{array}$$
    (10) $$\begin{array}{l} \varphi_{\text{bg}}\left(t_{\text{TE}}\right)=2\pi t_{\text{TE}}\cdot {\tilde{b}}_{\text{bg}} \end{array}$$
2. **Susceptibility Reconstruction (optionally in each iteration loop):** Maps of the magnetic susceptibility are obtained from a hybrid Tikhonov- and gradient-regularised minimisation approach based on de Rochefort et al. (2010) within *m*_EA_. In order to avoid any bias in the results due to additional magnitude conditioning, spatial priors are not used.

With each step, the raw phase is extended by the part of the EPR where *Q*_LC_ fulfils the threshold condition for “healthy data” following background phase subtraction (see **Figures 6, 10**). In accordance with the convergence observations made in the first numeric test runs, a total of five iterations were used.

#### 2.2.2. QSM Processing Without REFRASE Optimisation

The following steps were performed for conventional processing:

1. **Field Mapping:** Spatial unwrapping of the phase in each echo (Abdul-Rahman et al., 2007) within *m*_max_; temporal unwrapping in a central reference point; linear regression.
2. **Masking:** $m_{\text{EA}}\overset{!}{=}m_{\text{max}}$
3. **Background Field Correction:** Identification of spherical and dipole shaped field distortions using the multi-stage approach MUBAFIRE Lindemeyer et al. (2015)
4. **Susceptibility Reconstruction:** Same as REFRASE.

#### 2.2.3. Simulation

A simplified numerical model, containing a sphere-shaped *m*_max_ that mimics the observable brain support, is used to provide a proof-of-principle for REFRASE at ultra-high field (7T). The generated phantom with dimensions of 128 × 128 × 128 voxels represents a simplified human head and neck and contains air-filled cavities and a blood-filled bubble for the demonstration of strong field distortions. Furthermore, bone is introduced as a spherical shell inside the head structure. Typical susceptibility values, roughly adapted to values from Schenck (1996), Hopkins and Wehrli (1997), and Spees et al. (2001), were chosen for all tissue types: *χ*_Air_ = 0.36 ppm, *χ*_Skull_ = −0.9 ppm, *χ*_BloodBubble_ = −0.7 ppm, *χ*_Tissue_ = −9.0 ppm, including three spherical-shaped regions in the central brain imitating deep grey matter, *χ*_*R*_1__ = *χ*_Tissue_ + 0.2 ppm (hereafter serving as the control region), *χ*_*R*_2__ = *χ*_Tissue_ + 0.25 ppm, and *χ*_*R*_3__ = *χ*_Tissue_ + 0.3 ppm.

The field map was simulated based on fifth order SSHs using randomised coefficients. Gaussian random values with zero mean were assigned with standard deviation ranges of 1, 1, 2.5·10^−3^, 1.25·10^−4^, 1.25·10^−7^, and 1.25·10^−8^ for the zeroth to the fifth order, respectively. Field distortions generated by the numeric susceptibility model were included using dipole convolution (Marques and Bowtell, 2005), *b*_Dipole_ = *B*_0_·(*χ* * *d*) with *B*_0_ = 7 T, resulting in the overall field, *b* = *b*_SSH_ + *b*_Dipole_.

With a simplified, uniform *T*_2_ = 80 ms, $T_{2}^{*}$ was calculated as:

(11) $$\begin{array}{l} T_{2}^{*}=\frac{1}{\frac{1}{T_{2}}\text{+}∥\text{∇}b∥} \end{array}$$

within the brain, and 0 elsewhere. Based on the simulated field and the $T_{2}^{*}$-distribution, the true phase and the noise-free measurement signal were calculated based on

(12) $$\begin{array}{l} \varphi_{\text{true}}\left(t_{\text{TE}}\right)=2\pi b\cdot t_{\text{TE}} \end{array}$$

and

(13) $$\begin{array}{l} S\left(t_{\text{TE}}\right)=M_{0}\cdot {\text{e}}^{-\frac{t_{\text{TE}}}{T_{2}^{*}}}\cdot {\text{e}}^{\text{i}\cdot \varphi_{\text{true}}\left(t_{\text{TE}}\right)}\;. \end{array}$$

**Figure 3:**
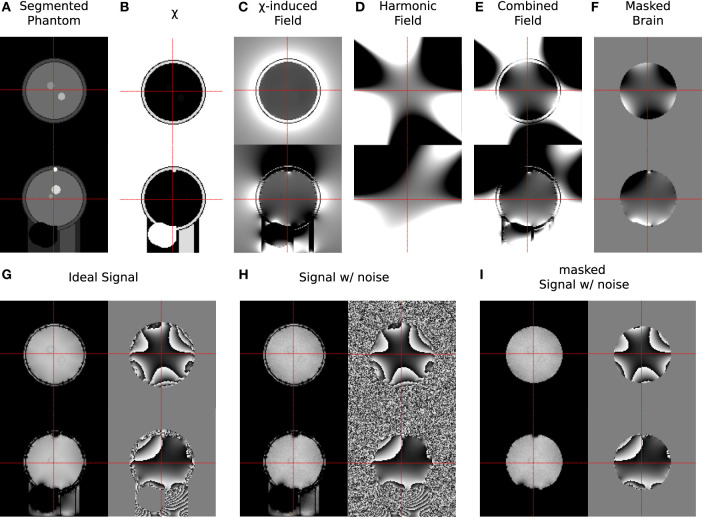
Samples from all stages of the simulation process showing transverse (upper) and sagittal (lower) slices. **(A)** Segmented regions in phantom; **(B)** assigned magnetic susceptibility; **(C)** field shift caused by susceptibility distribution; **(D)** harmonic field distortions; **(E)** combined field distortions; **(F)** field distortions within brain mask; **(G)** ideal signal (magnitude/phase); **(H)** noise-biased signal (magnitude/phase); **(I)** noise-biased signal within *m*_max_ (magnitude/phase). In each step the grey scale colour range was adapted to the contrast shown: **(A)** [0, 7]; **(B)** [−9.0, 0.36] ppm; **(C–F)** [−60, 60] Hz; **(G–I)** [0.5, 0.9] a.u. for magnitude and [−*π*, *π*] for phase, respectively.

Measurement conditions were simulated by adding complex Gaussian noise with a relative standard deviation of *σ*_noise_ = 0.01 to the signal, resulting in $\tilde{S}\left(t_{\text{TE}}\right)$. The signal was simulated for *t*_TE_ = [4, 16, 28, 40, 52] ms, with *M*_0_ set to 1 wherever non-air structures were present. Figure 3 illustrates the individual steps of the simulation procedure.

Conventional evaluation and REFRASE analysis with five iteration steps was conducted on the phase data obtained using a mask that contains the maximum extent of the simulated brain structure. This was repeated for different configurations of the *Q*_LC,min_ threshold between 0.4 and 0.9 applied to the phase of the second echo.

Susceptibility reconstruction was performed for each iteration step and each configuration using λ = 0.03 (Tikhonov) and *μ* = 0.001 (gradient regularisation). These regularisation values were determined heuristically, based on manual optimisation of image contrast vs. artefact level.

The resulting masks were then compared to the full brain mask of the numerical model, representing the maximum achievable coverage via the *relative difference in mask voxel count*:

(14) $$\begin{array}{l} n_{\text{rel}}=\frac{\sum \|m_{\text{max}}-m_{\text{EA}}\|}{\sum m_{\text{max}}} \end{array}$$

The mean susceptibility value, $\bar{\chi \left(R_{1}\right)}$, within control region *R*_1_ was determined in the reconstructed maps and its heterogeneity was assessed via the observed standard deviation, *σ*_*χ*_(*R*_1_).

A set of 10 numerical instances was simulated in a Monte Carlo-like approach and analysed. Each instance featured randomised parameters such as: positions of the air-filled cavities, the blood-bubble and differing coefficients of the harmonic background field characteristics.

#### 2.2.4. Measurements

**Figure 4:**
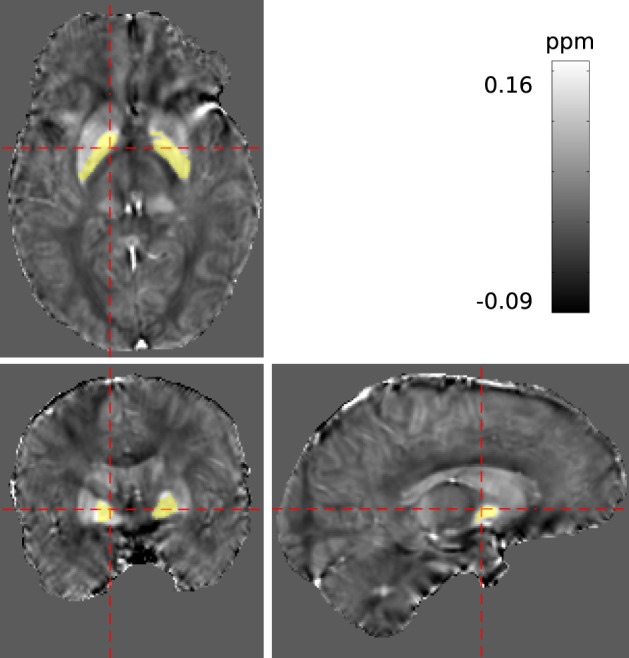
Segmentation of a potentially homogeneous region within the pallidum (PAL) highlighted in yellow, displayed in the reconstructed susceptibility maps.

A 3D multi-echo gradient echo sequence with monopolar readout, parametrised for two different studies, was acquired at 1mm isotropic resolution, FA = 11° and TR = 18 ms with a spatially-selective pulse. Having obtained written informed consent, measurements on a cohort of ten volunteers were conducted using a 4T MRI scanner with a single-channel birdcage coil. In total, three echoes were recorded at 2.5, 6.0, and 12.0 ms (with BW = [500, 500, 160] Hz/Px respectively), but only the first and last echo were processed in this study. A maximum coverage brain mask, *m*_max_, was derived using bet2 (FSL Toolkit, Smith, 2002). For each volunteer, bilateral regions within the globus pallidus of both hemispheres were manually segmented via ITKSnap Yushkevich et al. (2006) based on the susceptibility maps obtained. The segmentation is illustrated in Figure 4. All data underwent conventional evaluation as well as REFRASE analysis with five iterations. Regularisation parameters for QSM reconstruction were identical to the simulation. This was repeated for *Q*_LC,min_ threshold values between 0.4 and 0.9, applied to the phase of the second echo. Here, *n*_rel_ was calculated with reference to *m*_max_, and *σ*_*χ*_ and $\bar{\chi }$ were determined within the control region.

To demonstrate the merits of REFRASE one *in vivo* dataset was processed with STI Suite 3 (University of California, Berkeley; https://people.eecs.berkeley.edu/~chunlei.liu/software.html). V-SHARP (Li et al., 2011) was selected for background removal and StarQSM (Wei et al., 2015) was selected for susceptibility reconstruction. Three different configurations were computed: (a) using a classic FSL-bet2 brain mask, (b) using an eroded FSL-bet2 brain mask, and (c) using the REFRASE-preprocessed phase and mask.

## 3. Results

### 3.1. Simulation

**Figure 5:**
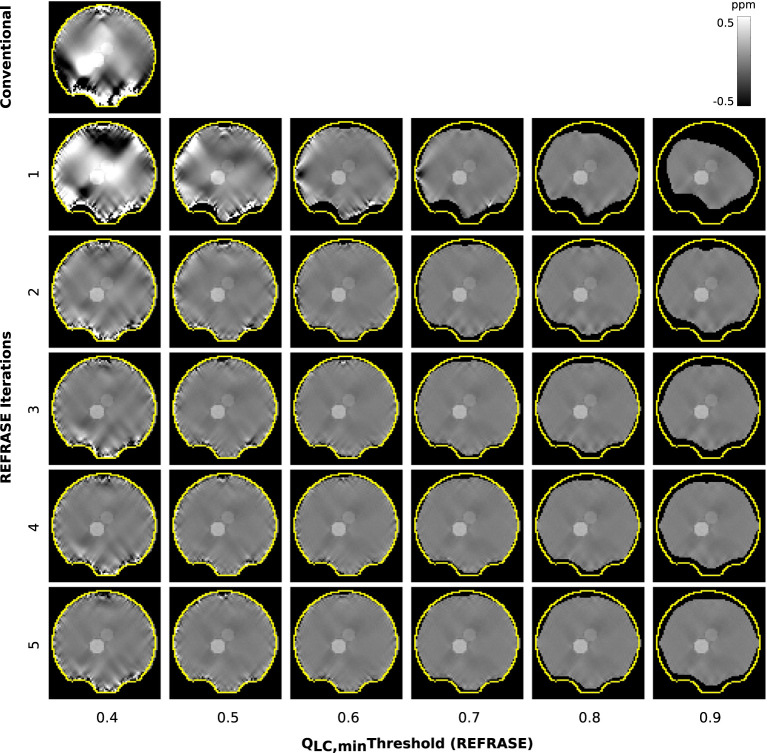
Overview of the susceptibility maps obtained in the simulation showing one coronal sample slice for each iteration (vertical) and each threshold configuration (horizontal). The top left image shows conventional reconstruction without REFRASE. Black background indicates the mask support of each illustrated image, while the yellow line indicates *m*_max_.

Figure 5 shows a sample slice of the susceptibility maps obtained using conventional and REFRASE processing. The conventional case shown in the top left, and particularly the first iteration step with the three lowest *Q*_LC,min_, clearly demonstrates how faulty or uncorrected field areas close to the rim of the mask distort the susceptibility result of the entire map, causing streaking artefacts. The homogeneity of the map increases with the iteration count and with higher threshold levels *Q*_LC,min_. However, it can be seen that very high thresholds result in reduced mask coverage.

**Figure 6:**
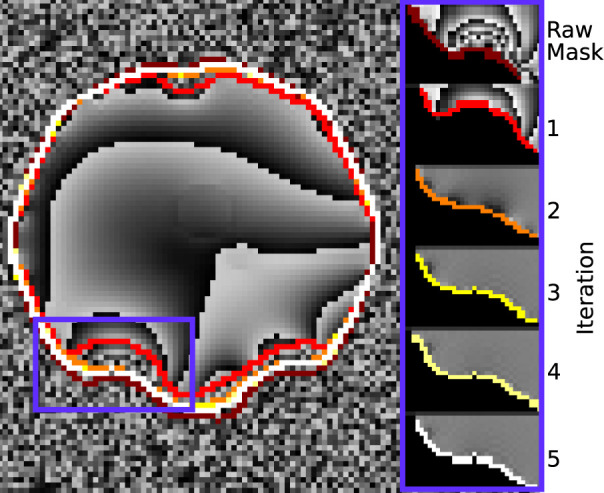
Illustration of the evaluation mask for conventional (raw) processing and REFRASE analysis on the synthetic data (coronal slice). The purple frame indicates the masked region shown on the right-hand side. For REFRASE, a threshold of *Q*_LC_ = 0.6 was chosen. Each coloured rim indicates the iteration step, shown in masked view on the right-hand side. The phase of the fourth echo is shown in the background.

Figure 6 demonstrates how the rim of the mask changes between conventional evaluation and each REFRASE iteration. Whereas the conventional mode naturally has full mask coverage, REFRASE approaches this solution with each iteration, while excluding noise-contaminated areas.

**Figure 7:**
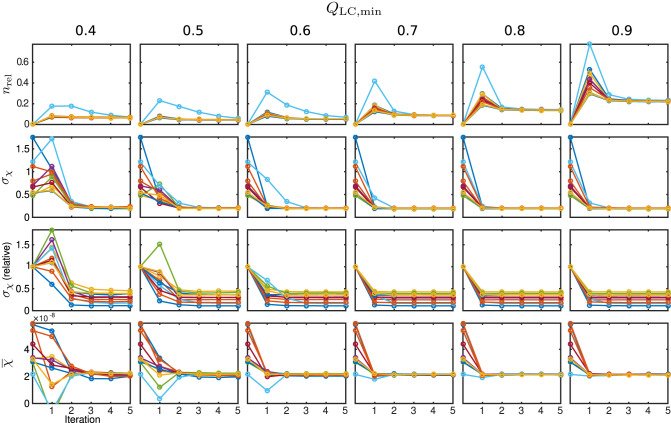
Susceptibility and mask results for the simulation. One individual colour is used for each Monte Carlo case. From the top row to bottom: relative mask difference between true mask and phase masking result (*n*_rel_); heterogeneity (standard deviation, *σ*_*χ*_) of susceptibility within reference area; heterogeneity normalised to conventional result without REFRASE; mean susceptibility value inside of reference area ($\bar{\chi }$). Each graph shows the conventional calculation without REFRASE as 0th point and the iteration result of REFRASE.

The behaviour of the relative mask difference and the susceptibility results are illustrated in Figure 7. The first row shows *n*_rel_. For all Monte Carlo cases, it can be seen that it significantly increases in the first iteration step but then decreases and approaches a constant level closer to zero, e.g., full coverage, with every further step. For *Q*_LC,min_ ≥ 0.7, the achievable constant difference minimum grows rapidly with the threshold level, implying a lower achievable coverage of *m*_max_. The absolute standard deviation within the control region *R*_1_, plotted in the second row, approaches a comparable minimum for all cases. However, a finite minimum is to be expected due to the artificially introduced noise level *σ*_noise_. The convergence only seems comparably slow for very low *Q*_LC,min_ = 0.4 and the standard deviation in iteration five lies slightly above the results for the higher *Q*_LC,min_. The relative standard deviation in comparison to the conventional approach, shown in the third row, supports this observation and makes the behaviour clearer for all Monte Carlo cases. With the exception of the first iteration of a few Monte Carlo cases, the standard deviation lies below that of the conventional analysis. As the heterogeneity should only be biased by noise, REFRASE generates the more precise estimate. The last row, illustrating the mean susceptibility value within *R*_1_, shows that the true value of $\bar{\chi }\left(R_{1}\right)=0.02\text{ppm}$ is well-approximated for *Q*_LC,min_ ≥ 0.6. For *Q*_LC,min_ = 0.5, the convergence is slightly slower, and for *Q*_LC,min_ = 0.4, the control region *R*_1_ is not well approximated after five iterations. Therefore, threshold values between 0.6 and 0.7 are a good trade-off between mask coverage and quality.

In order to gain a global insight into the distribution of reconstruction errors, an additional Root Mean Square Error (RMSE) analysis was performed for two different areas: Global RMSE, covering the full evaluation mask, *m*_EA_, and rim RMSE, defining a the rim of *m*_EA_ within six voxel widths from the mask surface. Since the presented QSM analysis and the data are reference-less, global constant offsets between result and simulated ground truth maps have been subtracted prior to RMSE calculation.

**Figure 8:**
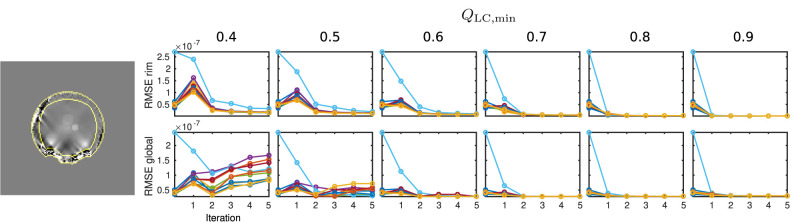
Results of the RMSE analysis on numeric data. On the left-hand side, the rim region delineation is visualised on a coronal sample slice from the numeric data (first iteration, *Q*_LC,min_ = 0.7), indicated by yellow lines. On the right-hand side, the results for global and rim RMSE are plotted for each computed value of *Q*_LC,min_ and each iteration. Conventional calculation without REFRASE is shown as the 0^*th*^ point. One individual colour is used for each Monte Carlo case.

The results of the RMSE analysis are displayed in Figure 8, showing a sample rim mask image and RMSE plots of the rim and global masks for all ten simulation cases.

### 3.2. Measurements

**Figure 9:**
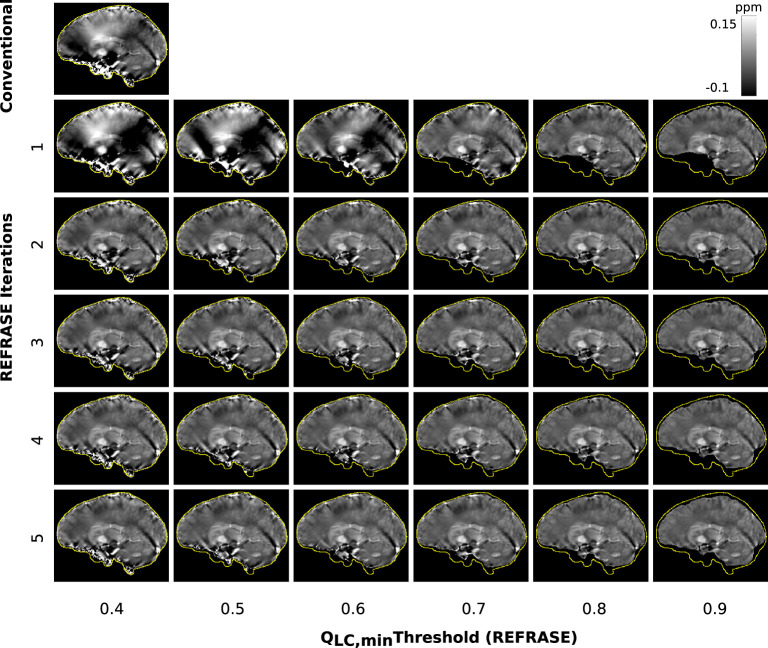
Overview of the susceptibility maps obtained from the measured data, showing one sagittal sample slice per iteration (vertical) and per threshold configuration (horizontal). The top left image shows conventional reconstruction without REFRASE. Black background indicates the mask support of each illustrated image, while the yellow line indicates *m*_max_.

Figure 9 shows susceptibility maps for one volunteer in a sample slice computed with the conventional approach and REFRASE. As observed in the numerical sample, the measurement in the first iteration also clearly shows the influence of faulty field areas at rim of the mask, distorting the susceptibility result of the entire map and the image centre by causing streaking artefacts as well as over- and underestimated areas. The homogeneity of the result increases with iteration count and with *Q*_LC,min_, while the mask difference appears to decrease.

**Figure 10:**
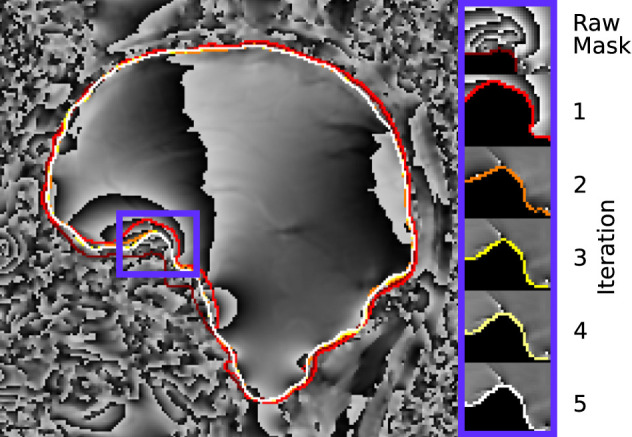
Illustration of the evaluation mask for conventional (raw) processing and REFRASE analysis on the measurement data (sagittal slice). The purple frame indicates the masked region shown on the right-hand side. For REFRASE, a threshold of *Q*_LC,min_ = 0.6 was chosen. Each coloured rim indicates the iteration step, shown in masked view on the right-hand side. The phase of the second echo is shown in the background.

Figure 10 demonstrates the changes near the rim of the mask between conventional evaluation and REFRASE with each iteration. Again, the results are comparable to the synthetic data. The REFRASE iterations approach *m*_max_ with each iteration, while noise-contaminated areas remain excluded.

**Figure 11:**
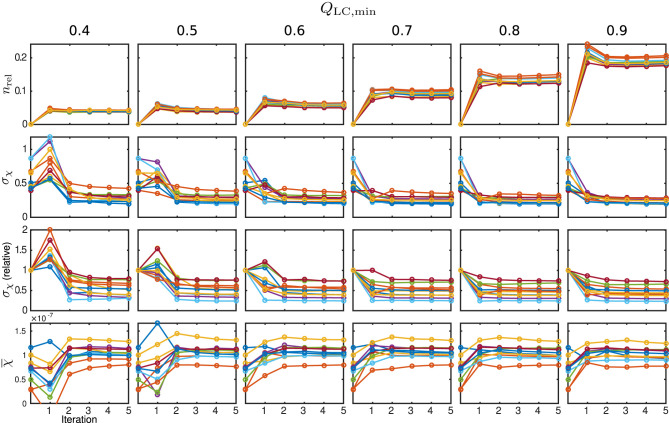
Susceptibility and mask results from *in vivo* measurements. One individual colour is used for each subject. From the top row to bottom: relative mask difference, *n*_rel_, between a mask from the classic approach and the REFRASE result; heterogeneity (standard deviation, *σ*_*χ*_) of susceptibility within reference area; heterogeneity relative to conventional result without REFRASE; mean susceptibility value ($\bar{\chi }$) inside reference area. Each graph shows the conventional calculation without REFRASE as 0th point and the iteration result of REFRASE.

The relative mask difference and the susceptibility results are illustrated in Figure 11. While *n*_rel_ again exhibits a peak in the first iteration of REFRASE, it decreases more slowly with each iteration compared to the synthetic data. This is potentially due to the higher complexity of the data. Nevertheless, a similar trend is observed. As was also the case in the simulation, a more rapid increase of minimum achievable *n*_rel_ appears from *Q*_LC,min_ ≥ 0.7 onward. In all cases, the absolute, as well as the relative, standard deviation within the segmented basal ganglia approach a value that is clearly lower than the conventional analysis. Leaving aside the first iteration, better homogeneity in the control region is confirmed for the acquired measurements from the second iteration onwards. Slow convergence is only observed for *Q*_LC,min_ = 0.4, similar to the numeric samples. The mean susceptibility, or rather the value it is converging to, naturally differs due to the individual physical properties of the volunteers. Even for the lowest *Q*_LC_, it shows convergence after four to five iterations. For *Q*_LC,min_ ≥ 0.6, the mean value curve already reaches steady behaviour after the second or third iteration.

**Figure 12:**
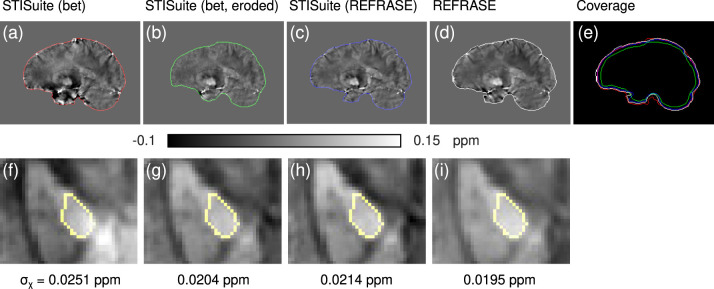
Comparison of different QSM reconstruction configurations: **(a,f)** STI Suite with FSL-bet2 mask, V-SHARP, and StarQSM; **(b,g)** STI Suite with eroded FSL-bet2 mask, V-SHARP, and StarQSM; **(c,h)** STI Suite with REFRASE phase and mask (*Q*_LC,min_ = 0.7, five iterations), V-SHARP and StarQSM; **(d,i)** REFRASE (same configuration) with MUBAFIRE and in-house QSM reco. Image **(e)** illustrates the masks of all methods. Row one **(a–e)** shows a sagittal sample slice from volunteer one, while row two **(f,g)** illustrates a magnified, transverse view of a sample slice indicating the analysed control region within the pallidum. Below each image, the standard deviation within the entire control region is printed, indicating the heterogeneity.

Figure 12 shows the results of the QSM processing comparison with STI Suite and REFRASE using our custom QSM reconstruction. The extent of the EA and the improvement of heterogeneity within the control region is also shown.

## 4. Discussion and Conclusions

A comparison between the behaviour of the relative mask difference, mean susceptibility, and homogeneity of the simulation with the *in vivo* measurements shows that the employed numeric model is a simplified, yet adequate representation of disturbances observed within actual measurements.

The results of the simulation confirm the functionality of REFRASE by showing clear convergence toward the known susceptibility values as well as demonstrating improved performance for the intra-region homogeneity within the control region, compared to the conventional analysis. The RMSE analysis of the rim shows better performance for all settings after five REFRASE iterations. However, while the global RMSE shows similar behaviour for strict thresholds, it increases significantly with iteration count for very weak thresholds (*Q*_LC,min_ = 0.4 or 0.5). This was to be expected as very low thresholds inevitably include unwanted EPRs. These noise-contaminated parts cannot be corrected for by REFRASE since they are not described by the physical model used for QSM and, hence, propagate into the entire map during QSM calculation. This explains the poorer convergence and homogeneity within the control region, and, consequently, here the results are closer to the conventional analysis.

In contrast, a very high *Q*_LC,min_ would provide excellent results within the central control regions, at the cost of substantial mask shrinkage. Nevertheless, even in this scenario, a convergent behaviour develops after a few iterations. Partial compensation for the mask shrinkage may be achieved by employing iteration counts much higher than those tested in the present study. However, results imply that when thresholding is too strict, regions with steep phase gradients will be irrevocably excluded. In summary, the highest *Q*_LC,min_ values are not suitable for high mask coverage and should only be chosen if the rim of the mask provides extremely poor phase quality and if the evaluation focusses on structures in the centre of the brain. Very low settings of *Q*_LC,min_ ≤ 0.5, on the other hand, are even less desirable as they tend to increase the reconstruction error.

The measurement-based results relating to mask difference and the intra-region homogeneity of the susceptibility maps obtained confirm the threshold values estimated in the simulation. This implies that even under realistic conditions, the proposed REFRASE scheme successfully improves stability within the control region and mask coverage of the results. The standard deviation in the control region has its lower limit at the tissue-specific heterogeneity. Thus, considering the physical principles of the processing steps applied, the decrease of *σ*_*χ*_ with iteration count is not due to a loss of contrast. Rather, it has to be implied by the larger spatial data support and by the exclusion of voxels with false field information, which would otherwise lead to artefact-induced heterogeneity.

As most other commonly used QSM background removal strategies at least partially shrink the mask to avoid edge effects, the correction of EPRs by conventional processing is not possible. This leads to either artefact contamination or, in case the EPRs are excluded in advance, implies a massive reduction of the *m*_EA_. While neither method can revert noise-induced phase falsification, Nyquist-exceeding phase gradients can be successfully recovered by using REFRASE (see Figure 10). This offers a valuable advantage, not only for dealing with typical artefacts generated by e.g., the nasal cavities but also for QSM applications in patients with brain tumours or traumatic injuries, especially near the surface of the brain. In both cases, bleeding or local aggregation of blood generate a susceptibility interface due to the iron content in the blood, which is much stronger than any usual intra-brain contrast and makes traditional evaluation in the surrounding areas difficult. While REFRASE is not able to recover the bleeding itself, it makes imaging the surrounding areas possible. However, care must be taken when applying REFRASE in subjects with bleeding inside of the brain rather than at the rim. Depending on the chosen parametrisation, such regions might be excluded by REFRASE, because they exhibit strong local phase gradients. Consequently, the investigation of a segmentation or preprocessing step to separate such regions from the brain support and from the exterior is an objective for further development.

To the best of our knowledge, only Topfer et al. (2015) and Buch et al. (2014) have presented rim phase correction approaches remotely comparable to REFRASE. Topfer et al. (2015) reported recovery limitations of one or two voxels closest to the mask edge as a result of employing first and second order derivatives. Even though our approach is not limited by a discrete kernel size or derivatives at a voxel level, the minimisation for an external susceptibility distribution, as employed in MUBAFIRE, causes overcompensation near the very edges of the mask and hence, REFRASE can, in its current form, also be affected by overcompensation in single voxels close to the outer rim. This issue was also reported by Liu et al. (2011a) in the original publication of the projection onto dipole fields (PDF) approach. The authors proposed the use of a dilated brain mask as *W*_*T*_, pushing the external susceptibility distribution further away from the boundary. Since this is identical to the REFRASE strategy, by using an EA smaller than *m*_max_, the scenario is much less likely to occur than in traditional PDF. While Buch et al. (2014) successfully demonstrated the modelling of distinct anatomic structures, such as the sinuses, and recovery of their effect on neighbouring tissue, the present approach represents a less source-specific, yet, more general means to recover phase data. A very recent publication from Buch et al. (2019) discusses the estimation of phase and susceptibility in the dural sinuses. Among other features, this approach utilises Taylor expansions of the phase to overcome signal voids. This nicely complements the rim phase recovery abilities of REFRASE by continuing the phase recovery beyond the brain surface.

To exclude additional bias introduced by the magnitude and to emphasise the advantages of REFRASE in a non-preconditioned scenario, spatial priors were deliberately excluded from all QSM analyses in this study. As a result, the QSM maps obtained are not as homogeneous and rich in contrast as common showcase maps with enforced contrast. Furthermore, as acquisition was via a single-channel birdcage, improvements in SNR by per-channel phase computation were not possible in the present study. However, our results also imply improvements for spatial-prior based QSM reconstruction.

The applied background identification steps, namely harmonic fields and external dipole sources from MUBAFIRE, were shown to perform very well on the processed datasets. Nevertheless, the newly introduced REFRASE principle can be applied with any other background removal strategy, as long as its field estimate can be estimated in or extrapolated to the maximum mask, *m*_max_. Alternatively, REFRASE-preprocessed phase can be fed into other QSM pipelines.

Finally, the QSM-Comparison showcase in Figure 12 outlines the importance of the phase quality at the mask rim for QSM reconstruction. Even a sophisticated QSM reconstruction performed on the inside of a maximised brain mask without restoration or exclusion of the rim phase, as in method (a), shows a heavy impact on the resulting QSM maps, even in central regions, as shown in subplot (f). While the homogeneity of the maps and within the control region generated with method (b) (eroded mask and V-SHARP/StarQSM) is significantly improved, the extent of the mask is clearly reduced. Evidently, method (c) (STI Suite with REFRASE preprocessing) and REFRASE with custom QSM produce the most robust maps with low control region heterogeneity, while keeping the mask coverage as high as possible.

It is important to note that the presented method used phase data from a birdcage coil and does not address the recombination of phase from multichannel receive arrays. However, REFRASE can be easily applied directly after an appropriate preprocessing step that combines the channels.

In conclusion, we have demonstrated a novel iterative data preparation method, REFRASE, which achieves significant improvements in terms of the quality of the susceptibility maps obtained and mask coverage for QSM. Both factors are significant for clinicians, as the quantitative precision of QSM is increased and the mask coverage becomes more comparable to that of other standard MRI contrasts. Furthermore, as ultra-high field MRI becomes more accessible, the method will evolve to its full potential, compensating for stronger local magnetic field gradients. Finally, the presented method is very valuable for low-resolution field mapping approaches that suffer from strong intra-voxel gradients. The free choice of the background-estimation method renders REFRASE a universal tool for arbitrary phase contrast applications.

## Data Availability Statement

The *in vivo* datasets for this manuscript are not publicly available due to ethical restrictions. Requests to access the datasets should be directed to the corresponding author: Prof. N. Jon Shah. The segmented susceptibility maps of the Monte Carlo set as well as the according result maps of conventional and REFRASE processing are available on FigShare (https://doi.org/10.6084/m9.figshare.12769853). Further, for the first Monte Carlo case, the maps of all simulation steps are included.

## Ethics Statement

The studies involving human participants were reviewed and approved by Ethics Committee of the Medical Faculty of the Rheinisch-Westfaelische Technische Hochschule Aachen (RWTH Aachen University, University Hospital, Aachen, Germany). The patients/participants provided their written informed consent to participate in this study.

## Author Contributions

JL conceived the presented idea, developed the theory and performed the computations, and wrote the manuscript. WW, AS, and JL planned, designed, and performed the measurements. NS provided equipment and infrastructure. JL, WW, AS, and NS discussed the method and results. All authors contributed to the final manuscript.

## Conflict of Interest

The authors declare that the research was conducted in the absence of any commercial or financial relationships that could be construed as a potential conflict of interest.

## Supplementary Material

The data used to generate the figures and to derive the presented results are contained in the supplementary archive file in form of spreadsheets.
